# Empirical Expression for AC Magnetization Harmonics of Magnetic Nanoparticles under High-Frequency Excitation Field for Thermometry

**DOI:** 10.3390/nano10122506

**Published:** 2020-12-14

**Authors:** Zhongzhou Du, Dandan Wang, Yi Sun, Yuki Noguchi, Shi Bai, Takashi Yoshida

**Affiliations:** 1 School of Computer and Communication Engineering, Zhengzhou University of Light Industry, Zhengzhou 450001, China; duzhongzhou1983@gmail.com (Z.D.); wangdandan0221@gmail.com (D.W.); 2 Department of Electrical and Electronic Engineering, Kyushu University, Fukuoka 819-0395, Japan; noguchi@em.ees.kyushu-u.ac.jp (Y.N.); t_yoshi@ees.kyushu-u.ac.jp (T.Y.); 3 School of Information Science and Engineering, Shenyang University of Technology, Shenyang 110870, China; stworldyy@gmail.com

**Keywords:** magnetic nanoparticle, Langevin function, Néel relaxation, Fokker–Planck equation, phase lag, magnetic nanoparticle thermometer

## Abstract

The Fokker–Planck equation accurately describes AC magnetization dynamics of magnetic nanoparticles (MNPs). However, the model for describing AC magnetization dynamics of MNPs based on Fokker-Planck equation is very complicated and the numerical calculation of Fokker-Planck function is time consuming. In the stable stage of AC magnetization response, there are differences in the harmonic phase and amplitude between the stable magnetization response of MNPs described by Langevin and Fokker–Planck equation. Therefore, we proposed an empirical model for AC magnetization harmonics to compensate the attenuation of harmonics amplitude induced by a high frequency excitation field. Simulation and experimental results show that the proposed model accurately describes the AC *M*–*H* curve. Moreover, we propose a harmonic amplitude–temperature model of a magnetic nanoparticle thermometer (MNPT) in a high-frequency excitation field. The simulation results show that the temperature error is less than 0.008 K in the temperature range 310–320 K. The proposed empirical model is expected to help improve MNPT performance.

## 1. Introduction

Magnetic nanoparticles (MNPs) have been widely studied for use in biomedical applications [1,2,3,4,5,6]. Magnetic nanoparticle-mediated hyperthermia (MNPH) [7,8,9,10] is a new anticancer therapy that heats local body parts to kill cancer cells using the difference in heat resistance between tumor tissue cells and normal cells. MNPs induce damage or necrosis of cancerous cells by elevating their temperature above 315–319 K (42–46 °C) without significantly harming the surrounding healthy tissue [11]. MNPH has received increasing attention from researchers because it is noninvasive and targeted, which are critical features for tumor therapy. It is crucial to accurately control the tissue temperature because it directly affects the curative effect of MNPH [10].

The magnetic nanoparticle thermometer (MNPT) [12,13,14,15,16,17] is a new tool that non-invasively measures temperature using the temperature dependency of the nonlinear magnetization of MNPs. J.B. Weaver et al. [12,13] experimentally validated the nonlinearity of the magnetization curve and used a fitted parameter to estimate the temperature. Liu et al. [14] investigated a theoretical model of MNP temperature measurement under a DC magnetic field, which laid the foundation for developing MNP temperature measurement technology. In previous work [17], we proposed and demonstrated a temperature measurement and control system using MNPs that achieved an error of less than 0.5 K at a target temperature of 315 K, showing the feasibility of the method. However, the frequency of the excitation field heating the MNPs reached 20 kHz. An improved temperature model could be used to apply a low-frequency excitation field (usually less than 1 kHz). Therefore, an additional exciting coil is needed to produce a low-frequency excitation field for temperature measurement. Furthermore, the magnetic nanoparticle sample moves between the heating and exciting coils through a mechanical device, making the MNPT setup complicated.

In these previous studies, the theoretical models for temperature measurement were based on the Langevin function, which describes the static magnetization of an MNP ensemble. There are always rotational Brownian and Néel relaxations in MNPs exposed to an AC excitation field [18,19]. The Langevin function is only valid in an equilibrium (or static) state and does not accurately describe MNP magnetization dynamics when MNP relaxation cannot be neglected. These are particularly problematic in an MNPT; i.e., MNPT application is restricted to a low-frequency excitation field, and an additional and complicated temperature setup is necessary. An MNP magnetization model for a higher-frequency excitation field is needed to expand MNPT application to such a field without a complicated setup.

The Fokker–Planck equation accurately describes AC magnetization dynamics dominated by Néel relaxation. In this study, we investigated the stable AC magnetization described by the Fokker-Planck equation and the Langevin function, and found that there are differences in the harmonic phase and amplitude between the stable magnetization response of MNPs described by Langevin and Fokker–Planck equation. We studied harmonic amplitude and phase dependences on Néel relaxation to derive simple empirical models for harmonic magnetization. Moreover, we investigate the temperature error on the basis of the proposed empirical harmonic model for an MNPT in a high-frequency excitation field.

## 2. Model and Methods

### 2.1. Langevin Function

When a low-frequency excitation field is applied in which MNP relaxation is negligible, the MNP magnetization is described using the Langevin function:

(1) $$M_{L}=M_{s}L\left(\xi \right)$$

where L(ξ)=coth(ξ)−1/ξ is the Langevin function, *ξ* = *μ*_0_*mH*/*k_B_T*, *H = H*_0_sin(2π*ft*) is the AC excitation magnetic field, *f* is the frequency of the applied field, *k_B_* is the Boltzmann constant, *T* is the absolute temperature, and *m* is the magnetic moment.

Expanding Equation (1) in a Taylor series and consolidating similar items on a frequency basis allows *M_L_* to be expressed as:

(2) $$M_{L}(t)=\sum_{j=1}^{n}A_{2j-1}\sin\left(\left(2j-1\right)2\pi ft\right)$$

where *A*_2*j*−1_ is the amplitude of the (2*j*−1)-th harmonic magnetization. The Maclaurin expansion can be used to express the harmonic amplitude *A*_2*j*−1_ as follows:

(3) $$\{\begin{array}{c} \begin{array}{l} A_{1}=M_{s}\left(\frac{\chi }{3}-\frac{\chi^{3}}{60}+\frac{\chi^{5}}{756}-\frac{\chi^{7}}{8640}+\frac{\chi^{9}}{95040}+\cdots \right)\\ A_{3}=M_{s}\left(\frac{\chi^{3}}{180}-\frac{\chi^{5}}{1512}+\frac{\chi^{7}}{14400}+\cdots \right)\\ A_{5}=M_{s}\left(\frac{\chi^{5}}{7560}+\frac{\chi^{7}}{43200}+\cdots \right) \end{array}\\ \vdots \end{array}$$

where $\chi =\frac{\mu_{0}H_{0}m}{k_{B}T}$.

We can obtain the expression of harmonic magnetization using the Langevin function to describe MNP magnetization in a low-frequency excitation field. Researchers can then use the analytical expression to extend application of this magnetization to measuring temperature and estimating core size distribution. However, the Langevin function cannot accurately describe the AC magnetization of MNPs considering relaxation in a high-frequency excitation field.

### 2.2. Fokker–Planck Equation for Néel Relaxation

When Néel relaxation is significant, the dynamics of single-domain spherical MNPs can be accurately described by the Fokker–Planck equation. Assuming that the magnetic anisotropy energy is uniaxial and the easy axes of all the particles are parallel to the excitation field, the Fokker–Planck equation for Néel relaxation is [20,21]:

(4) $$2\tau_{N0}\frac{\partial W\left(\theta ,t\right)}{\partial t}=\frac{\partial }{\partial x}\left[(1-x^{2})\left(\frac{\partial W\left(\theta ,t\right)}{\partial t}-\xi \left(t\right)W\left(\theta ,t\right)-\alpha_{K}xW\left(\theta ,t\right)\right)\right]$$

where *x* = cos*θ*, $\alpha_{K}\equiv \frac{2KV_{c}}{k_{B}T}$, $\tau_{N0}\equiv \frac{m\left(1+{\alpha^{\prime }}^{2}\right)}{2k_{B}T\gamma \alpha^{\prime }}$ is the Néel relaxation time, $\alpha^{\prime }$ is the damping coefficient, γ is the electron gyromagnetic ratio, *K* is the anisotropy constant, *θ* is the angle of the magnetic moment *m* with respect to the excitation field *H*, *W*(*θ*, *t*) is the distribution function of *m*, *M_s_* = *m*/*V_c_* is the saturation magnetization, and *V_c_* is the MNP volume.

We numerically solve Equation (4) by expanding *W*(*θ*, *t*) in terms of Legendre polynomials:

(5) $$W\left(\theta ,t\right)=\sum_{n=0}^{\infty }a_{n}\left(t\right)P_{n}\left(\cos\theta \right)$$

where *a_n_*(*t*) is the time-dependent coefficient of the *n*-th order spherical harmonic, and *P_n_*(cos*θ*) is the *n*-th order Legendre polynomial. Combining Equations (4) and (5), we obtain:

(6) $$2\tau_{N0}\sum_{n=0}^{\infty }P_{n}\frac{da_{n}}{dt}=\sum_{n=0}^{\infty }a_{n}\left\{\frac{d}{dx}\left[\left(1-x^{2}\right)\left(\frac{dPn}{dx}-\xi \left(t\right)P_{n}-\alpha_{K}xP_{n}\right)\right]\right\}$$

Standard recursion relations give the following set of coupled ordinary differential equations for *a_n_*(*t*):

(7) $$\begin{array}{cc} \frac{2\tau_{N0}}{n\left(n+1\right)}{\dot{a}}_{n}= & -a_{n}+\xi \left(t\right)\left(\frac{a_{n-1}}{2n-1}-\frac{a_{n+1}}{2n+3}\right)\\ & +\alpha_{K}[\frac{n-1}{\left(2n-3\right)\left(2n-1\right)}a_{n-2}+\frac{1}{\left(2n+1\right)\left(2n+3\right)}a_{n}\\ & -\frac{n+2}{\left(2n+3\right)\left(2n+5\right)}a_{n+2}] \end{array}$$

The distribution function *W*(*θ*, *t*) can be obtained from *a_n_*. The magnetization *M_FP_* in the direction of the excitation field can be calculated with the following equation:

(8) $$M_{FP}=M_{s}\int_{0}^{\pi }W\sin\theta \cos\theta d\theta$$

Expanding Equation (8) in a Fourier series and consolidating similar items on a frequency basis allows *M_FP_* to be expressed as:

(9) $$M_{FP}=\sum_{j=1}^{n}C_{2j-1}\sin\left(\left(2j-1\right)\omega t+\varphi_{2j-1}\right)$$

where *C*_2*j*−1_ and *φ*_2*j*−1_ are the amplitude and phase of the (2*j*−1)-th harmonic magnetization, respectively. Note that an analytical expression for *a_n_* cannot be obtained, so *C*_2*j*−1_ and *φ*_2*j*−1_ are numerically calculated.

The Fokker-Planck function can describe accurately AC magnetization dynamics (dominated by Brownian rotational relaxation). However, the model for describing AC magnetization dynamics of MNPs based on Fokker-Planck equation is very complicated and the numerical calculation of Fokker-Planck function is time consuming, and the analytical harmonic expression for MNPT is hard to obtain. The Langevin function is a simple model for describing MNPs magnetization response with no considering of Néel relaxation, and the analytical harmonic expression is shown in Equation (3). Therefore, we try to construct an empirical model for AC magnetization harmonics affected by the Néel relaxation.

### 2.3. Compensation Expression for MNP Magnetization Harmonics

We performed simulations to analyze the difference in stable AC magnetization between the Fokker–Planck equation and Langevin function and studied the harmonic amplitude and phase dependences on Néel relaxation time. In the simulations, the excitation field had an amplitude of 1 mT and a frequency of 20 kHz. The MNP ensemble magnetization based on the Langevin function was calculated via Equation (1), and that based on the Fokker–Planck equation was numerically calculated via Equation (8) under different Néel relaxation times (*τ_N_*_0_ = 10 ns, 5 ns, and 1 ns). Utilizing cross-correlation principle, digital phase-sensitive detection algorithm (DPSD) can extract effectively the amplitude and phase of signal to be measured from noise [22]. We obtained the harmonic amplitudes and phases of the ensemble magnetization via DPSD.

**Figure 1:**
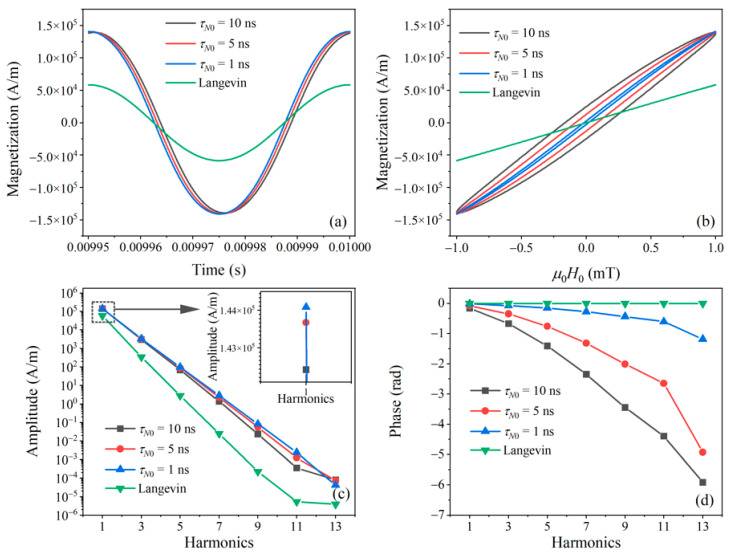
MNP magnetization response calculated with the Langevin function and Fokker–Planck equation under different Néel relaxation times (*τ_N_*_0_ = 10 ns, 5 ns, and 1 ns). (**a**) The MNP magnetization response. (**b**) *M*–*H* curves. The harmonics (**c**) amplitude and (**d**) phase. The excitation field has an amplitude of 1 mT and a frequency of 20 kHz. The parameters for these simulations are *d_c_* = 25 nm, *T* = 297 K, *K* = 4 kJ/m^3^, *M_s_* = 300 kA/m, $\alpha^{\prime }$ = 0.1, and γ = 1.75 × 10^11^ rad/s T.

As shown in Figure 1a, the difference between the magnetization responses calculated from the Langevin function and Fokker–Planck equation is mainly in the amplitude and time delay. Figure 1b shows the AC *M*–*H* curves of the MNPs. The Langevin-based *M*–*H* curve has no hysteresis because relaxation is neglected. The Fokker–Planck-based magnetization, however, has a hysteresis loop in the *M*–*H* curves, which indicates that the MNP magnetization response with Néel relaxation delays the excitation field. The delay increases with increasing Néel relaxation time. As shown in Figure 1c, the harmonic amplitude decays exponentially with the harmonic number. The harmonic amplitude of the Fokker–Planck equation is greater than that of the Langevin function, which is because all the easy axes are assumed to be parallel to the excitation field when using the Fokker–Planck equation. Figure 1d presents the harmonic phase of MNP magnetization. The Fokker–Planck results show that the harmonic phase lag increases for a higher harmonic order. For the same harmonic order, the phase lag increases with Néel relaxation time.

Though the Fokker–Planck equation can accurately describe AC magnetization dynamics, an analytical harmonic expression cannot be obtained. We investigated the difference in AC magnetization between the Fokker–Planck equation and Langevin function to obtain an empirical model for MNP magnetization harmonics.

As shown in Figure 1, the harmonic magnetization calculated with the Fokker–Planck equation differs from that of the Langevin function in harmonic amplitudes and phases. We use the following expression to compensate the difference:

(10) $${M_{FP}|}_{2j-1}=G_{2j-1}\cdot A_{2j-1}\sin\left(\left(2j-1\right)2\pi ft+\varphi_{2j-1}\right)$$

where ${M_{FP}|}_{2j-1}$ is the (2*j*−1)-th harmonic magnetization, *G*_2*j*−1_ is the compensation function, *φ*_2*j*−1_ is the phase, and *A*_2*j*−1_ is the harmonic amplitude calculated with the Langevin function. Using *G*_2*j*−1_ and *A*_2*j*−1_, the harmonic amplitude *C*_2*j*−1_ of MNP magnetization based on the Fokker–Planck equation can be expressed as follows:

(11) $$\{\begin{array}{c} \begin{array}{l} C_{1}=G_{1}\cdot A_{1}=G_{1}\cdot M_{s}\left(\frac{\chi }{3}-\frac{\chi^{3}}{60}+\frac{\chi^{5}}{756}-\frac{\chi^{7}}{8640}+\frac{\chi^{9}}{95040}+\cdots \right)\\ C_{3}=G_{3}\cdot A_{3}=G_{3}\cdot M_{s}\left(\frac{\chi^{3}}{180}-\frac{\chi^{5}}{1512}+\frac{\chi^{7}}{14400}+\cdots \right)\\ C_{5}=G_{5}\cdot A_{5}=G_{5}\cdot M_{s}\left(\frac{\chi^{5}}{7560}+\frac{\chi^{7}}{43200}+\cdots \right) \end{array}\\ \vdots \end{array}$$

Note that we propose empirical expressions only for harmonic amplitudes because they are used in the MNPT for a high-frequency excitation field, which will be presented later.

## 3. Simulation

We performed simulations to verify the feasibility of the compensation expression for harmonic amplitude in Equation (11). The compensation function *G* is associated with many parameters such as temperature and excitation field. To simplify the function *G*, we investigated its dependence on the excitation field strength. In this simulation, the MNP sample was exposed to an AC excitation field *H* = *H*_0_cos(2π*ft*), where *H*_0_ was set from 1 to 15 mT with a step of 1 mT and *f* was set at 20 kHz.

**Figure 2:**
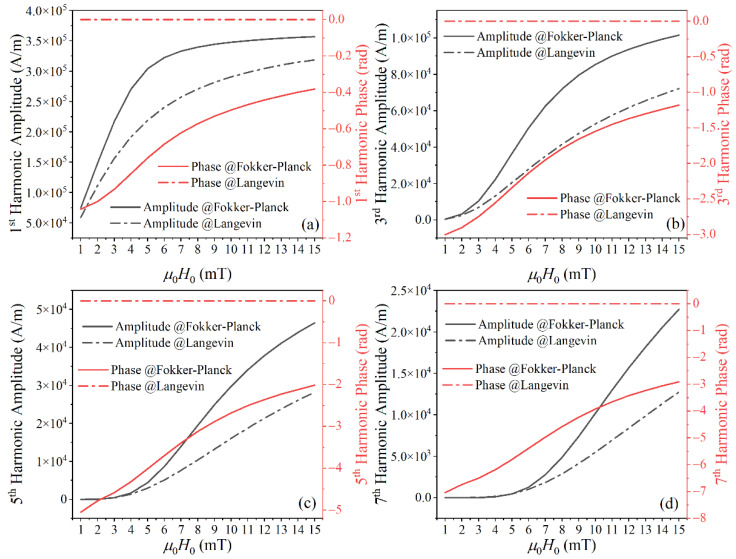
The (**a**) 1st, (**b**) 3rd, (**c**) 5th, and (**d**) 7th harmonic amplitudes and phases of MNP magnetization calculated with the Langevin function and Fokker–Planck equation under different excitation field strengths. The parameters for these simulations are *d_c_* = 25 nm, *T* = 297 K, *K* = 4 kJ/m^3^, *M_s_* = 300 kA/m, $\alpha^{\prime }$ = 0.1, and *γ* = 1.75 × 10^11^ rad/s T.

As shown in Figure 2a, the difference between the Fokker–Planck equation and Langevin function for the first harmonic increased with *H*_0_ for low *H*_0_ and decreased with *H*_0_ for high *H*_0_. Because higher harmonics require greater *H*_0_ to reach saturation, the differences in higher harmonics keep increasing with *H*_0_ in the range of *H*_0_ investigated, shown in Figure 2b–d. The harmonic phase lag (−*φ*_2*j*−1_) decreases with increasing excitation field. The phase lag of the harmonic becomes large for higher harmonics.

**Figure 3:**
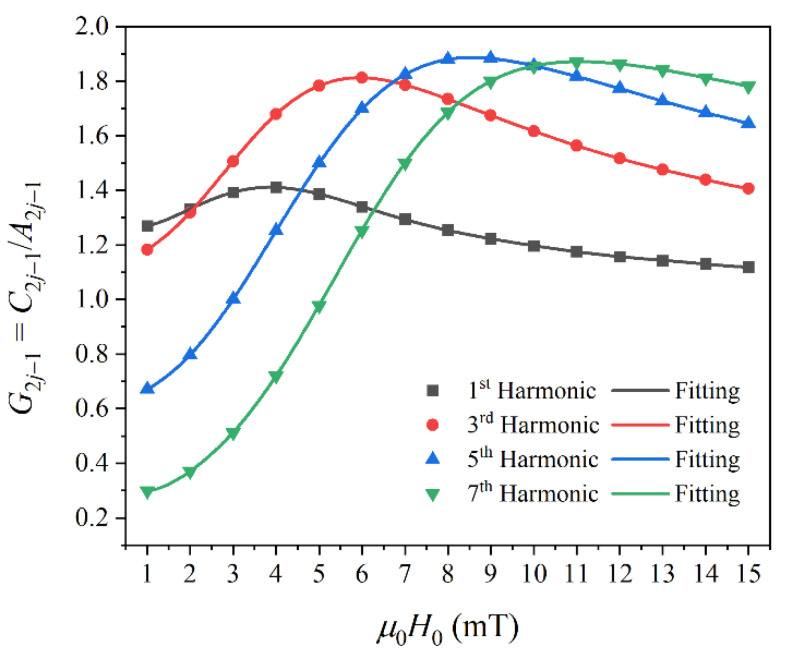
Dependence of the compensation function *G*_2*j*−1_ = *C*_2*j*−1_/*A*_2*j*−1_ on the excitation field strength *H*_0_.

According to the proposed compensation expression in Equation (11), we investigated the dependence of *G* on *H*_0_. Figure 3 shows the dependence of *G*_2*j*−1_ on *H*_0_. The symbols represent *G*_2*j*−1_ calculated with *G*_2*j*−1_ = *C*_2*j*−1_/*A*_2*j*−1_, and the solid lines represent polynomial curve fits given by:

(12) $$G_{2j-1}(H)=a_{2j-1,0}+\sum_{n=1}^{N}a_{2j-1,n}H^{n}$$

**Figure 4:**
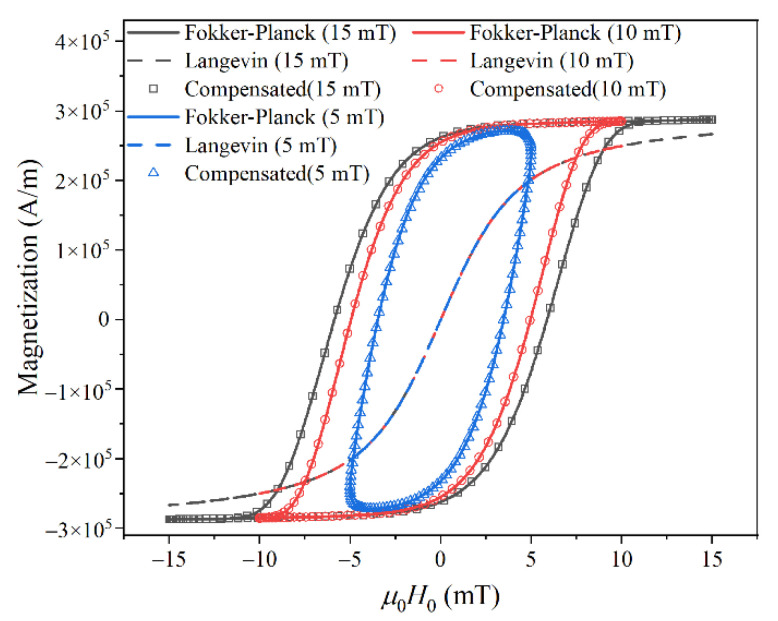
Reconstructed MNP magnetization response based on a compensation model.

Then, using the fitted compensation function *G*_2*j*−1_ and harmonic phase, we reconstructed the MNP magnetization response based on Equation (10). As shown in Figure 4, the reconstructed AC *M*–*H* curve at each *H*_0_ nicely fits that calculated from the Fokker–Planck equation.

## 4. Experiment and Results

In the experiments, the iron oxide nanoparticles (Fe_3_O_4_) called SHP-20 (SHP-20, Ocean NanoTech, San Diego, CA, USA) were used as MNP samples. SHP-20 consists of iron oxide nanoparticles with a carboxylic acid group and has an iron concentration of 5 mg (Fe)/mL. The solvent of the sample is deionized H_2_O with 0.03% NaN_3_. The effective core diameter of SHP-20 was 20 nm. The MNP sample was immobilized with an epoxy resin to avoid the effect of Brownian rotational relaxation. The sample was placed in a DC excitation field with a strength of 50 mT during the immobilization process, ensuring the easy axes of MNPs aligned along the same direction.

**Figure 5:**
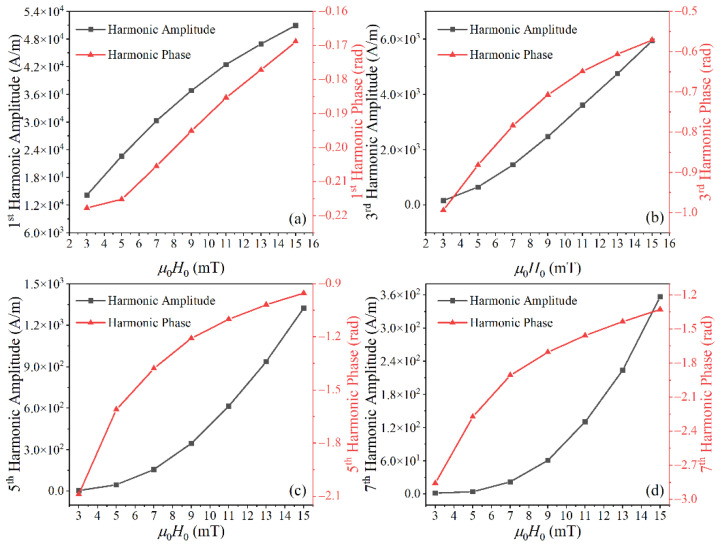
Experimental results for the (**a**) 1st, (**b**) 3rd, (**c**) 5th, and (**d**) 7th harmonic amplitudes and phases of MNP magnetization.

Using equipment constructed in a laboratory [23], the saturation magnetization (211 kA/m) of the MNP sample was determined under a static magnetic field with a strength of 1 T. The MNP sample was exposed to an AC excitation field and placed so that all the easy axes were parallel to the direction of the AC excitation field. The strength of the AC excitation field, *H*_0_, was set from 3 to 15 mT with a step of 2 mT at a frequency of 20 kHz. The temperature of the MNP sample was controlled at 297 K. We obtained the harmonic amplitudes (*C*_2*j*−1_) and phase (*φ*_2*j*−1_) of magnetization at each *H*_0_. As seen from Figure 5, the harmonic amplitudes increased with *H*_0_, and the phase lag (–*φ*_2*j*−1_) decreased with increasing *H*_0_. The higher the harmonic order is, the greater the harmonic phase is; i.e., the Néel relaxation has greater influence on the phase of higher harmonics.

**Figure 6:**
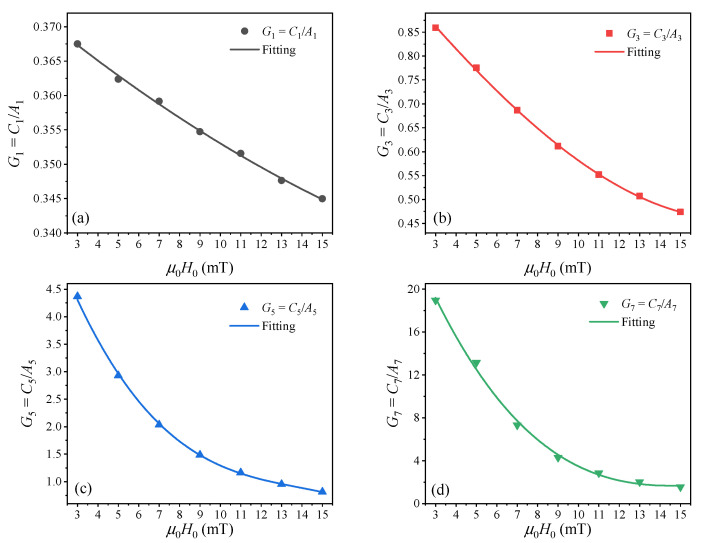
Experimental results for compensation function (**a**) *G*_1_ = *C*_1_/*A*_1_, (**b**) *G*_3_ = *C*_3_/*A*_3_, (**c**) *G*_5_ = *C*_5_/*A*_5_, and (**d**) *G*_7_ = *C*_7_/*A*_7_.

Then, we calculated the magnetization of the MNP sample using the Langevin function and obtained the harmonic amplitudes *A*_2*j*−1_. The effective core diameter of SHP-20 was set at 20 nm. Therefore, the function *G*_2*j*−1_ = *C*_2*j*−1_/*A*_2*j*−1_ associated with *H*_0_ can be obtained. In Figure 6, the symbols represent *G*_2*j*−1_ for different values of *H*_0_, and the solid lines represent polynomial curve fits using Equation (12).

**Figure 7:**
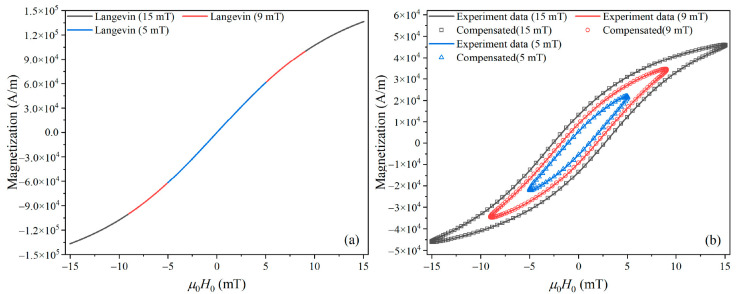
(**a**) AC *M*–*H* curves calculated from the Langevin function with a core diameter of 20 nm. (**b**) Reconstructed MNP magnetization response based on a compensation model.

We used Equation (11) to compensate the difference in harmonic amplitude caused by Néel relaxation. Then, using the fitted compensation function *G*_2*j*−1_ and harmonic phase, we reconstructed the MNP magnetization response using Equation (10). The reconstructed AC *M*–*H* curves match well with the experimental results, as shown in Figure 7.

## 5. Magnetic Nanoparticle Thermometry at High Frequency

In a previous study, a harmonic amplitude–temperature MNPT model was constructed using the Langevin function at a low frequency (less than 1 kHz) [16]. When a high-frequency excitation field is used, the Néel relaxation affects the harmonic amplitudes of MNP magnetization as discussed above.

We now use the empirical model of harmonic amplitude in Equation (11) to introduce the harmonic amplitude–temperature model of magnetic nanoparticle thermometry under Néel relaxation. In this harmonic amplitude–temperature model, we use the first and third harmonic amplitudes:

(13) $$\left\{ \begin{array}{l} C_{1\_meas}/G_{1}=M_{s}\left(\frac{\hat{\xi }}{3}-\frac{{\hat{\xi }}^{3}}{60}+\frac{{\hat{\xi }}^{5}}{756}-\frac{{\hat{\xi }}^{7}}{8640}+\frac{{\hat{\xi }}^{9}}{95040}+\cdots \right)\\ C_{3\_meas}/G_{3}=M_{s}\left(\frac{{\hat{\xi }}^{3}}{180}-\frac{{\hat{\xi }}^{5}}{1512}+\frac{{\hat{\xi }}^{7}}{14400}+\cdots \right) \end{array}\right.$$

where $\hat{\xi }=\frac{\mu_{0}mH_{0}}{k_{B}\hat{T}}$, $\hat{T}$ is the estimated temperature, and *C*_1_*meas*_ and *C*_3_*meas*_ are the measured first and third harmonic amplitudes.

**Figure 8:**
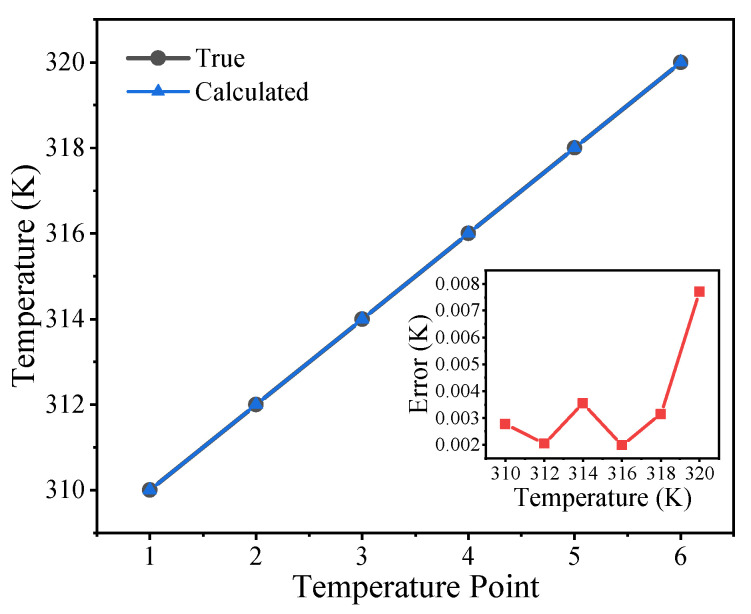
Simulation results of temperature estimation for an MNPT under Néel relaxation. The inset shows the temperature errors.

We performed simulations to verify the temperature determined using the empirical expression of harmonic amplitudes in Equation (11). In the simulations, the absolute temperature *T* was changed from 310 to 320 K with a step of 2 K. The AC excitation magnetic field had an amplitude of 2 mT and a frequency of 100 kHz. The saturation magnetization and anisotropy constant of the MNP sample were set at 200 kA/m and 4 kJ/m^3^, respectively. The MNP sample had a normal core diameter of 20 nm without a core size distribution. The temperature was calculated from Equation (13) using the Levenberg–Marquardt algorithm, and then the true temperature was subtracted to give the temperature errors. As shown in Figure 8, the error increased with temperature. Although the magnetization response decreased with increasing temperature and the signal-to-noise ratio was low at high temperature, the maximum temperature error was less than 0.008 K in the temperature range 310–320 K.

## 6. Conclusions

We studied the stable AC magnetization described by the Fokker–Planck equation (dominated by Néel relaxation) and Langevin function. We proposed a simple, empirical harmonic model. Simulation and experimental results showed that the proposed empirical model accurately describes AC harmonic magnetization, and the AC *M*–*H* curve constructed with the proposed empirical model matches well with the measured results. Moreover, we proposed a harmonic amplitude–temperature model for an MNPT under Néel relaxation in a high-frequency excitation field. The simulation results showed a temperature error of less than 0.008 K at the MNPH frequency level. The empirical harmonic model is expected to help improve the performance of MNPTs and extend their applications in MNPH.
